# Effect of Body Condition Score, Treatment Period and Month of the Previous Lambing on the Reproductive Resumption of Melatonin-Treated Sarda Breed Sheep during Spring

**DOI:** 10.3390/ani11071898

**Published:** 2021-06-25

**Authors:** Luisa Pulinas, Giovanni Cosso, Maria Consuelo Mura, Melissa Carvajal-Serna, Hatem Ouled Ahmed, Vincenzo Carcangiu, Sebastiano Luridiana

**Affiliations:** 1 Department of Veterinary Medicine, University of Sassari, Via Vienna, 2, 07100 Sassari, Italy; luisapulinas@hotmail.it (L.P.); gicosso@uniss.it (G.C.); mcmura@uniss.it (M.C.M.); endvet@uniss.it (V.C.); 2 Instituto Universitario de Investigación en Ciencias Ambientales de Aragón (IUCA), Universidad de Zaragoza, 50013 Zaragoza, Spain; melissac@unizar.es; 3 Laboratoire d’Analyses Genetiques Animals, Institut de la Recherche Vétérinaire de Tunisie, 20 Rue Djebel Lakhdar La Rabta, Tunis 1006, Tunisia; ouledahmedh@yahoo.fr

**Keywords:** melatonin treatment, Sarda breed sheep, reproductive performances

## Abstract

**Simple Summary:**

Improving reproductive efficiency in sheep farms is a crucial task for researchers. Therefore, the present research considers the conditions commonly found in Sarda sheep farms and evaluates the effects on reproductive activity via the following factors: exogenous melatonin treatment through subcutaneous implants, different periods of melatonin treatment, varying body condition scores (BCS) and the previous lambing of the treated ewes. The results of the present research suggest that melatonin treatment is able to reliably enhance reproductive efficiency. Furthermore, the findings indicate that optimal reproductive efficiency can be achieved by ensuring that melatonin is administered in April to ewes that have a BCS of >2.5 and have passed their third month of lactation.

**Abstract:**

Stakeholders place great emphasis upon rationalizing the management and rearing techniques which are utilized within sheep farms. The present study aimed to investigate factors which may improve the reproductive performance of melatonin-treated Sardinian sheep via a series of three trials. The first trial (*n* = 100) investigated the effect of melatonin treatment alongside body condition score (BCS), the second trial (*n* = 150) investigated the effect of treatment alongside the date of treatment (treatment period) and the third trial (*n* = 150) investigated the effect of treatment alongside the previous lambing of the ewes. The findings indicated that melatonin is an effective tool for anticipating and improving the reproductive activity of in Sarda breed sheep during the springtime. Furthermore, to obtain optional results, melatonin implantation should be conducted in April, in ewes that have a BCS of >2.5 and that have passed their third month of lactation.

## 1. Introduction

Small ruminants reared in the Mediterranean area show reproductive seasonality via recurrent physiological alternations of reproductive activity and sexual rest, which are linked to the photoperiod trend [1]. The breeding season of this species usually begins during the summer or early fall, in response to the shortening of the day length and ends during the late winter or early spring [2]. The perception of light and dark alternation affects the epiphysis secretion of melatonin, with high levels being secreted during night-time hours and low levels during daylight hours [3]. Melatonin is considered to be the organic informer of the photoperiod trend and the main regulator of reproductive seasonality [4]. Research has advocated for the administration of melatonin to improve reproductive efficiency in a variety of sheep breeds and subcutaneous implants have been the most commonly utilized method of doing so [5,6,7]. The melatonin released by the implants is able to mimic short days, without causing an inhibition of natural melatonin secretion, thus stimulating reproductive activity in sheep [8]. For dairy sheep within the Mediterranean area, lambing at the beginning of autumn is a crucial requirement for enabling six to seven months of lactation to occur [9]. Therefore, to allow for autumn lambing, sheep should mate in spring, during their anestrus period. During the springtime, however, despite the sheep being adequately sensitized to long days, reproductive activity is less stimulable and the effects of melatonin treatment do not provide the expected results [10]. This effect may be linked to several factors that derive from the careless management of the animals before they are included in the breeding plans. Firstly, the inclusion of animals that are in the ascending phase of lactation could affect reproductive recovery in spring because their energy is primarily used for milk production rather than for other physiological activities such as reproduction [9]. Another factor influencing the non-optimal responses to melatonin treatment may be the body condition score (BCS), as research has highlighted that animals with low BCS may have low reproductive efficiency [7]. Further factors influencing reproductive responses may be the period of administration of melatonin and the subsequent introduction of the males. As a result, the effect of the melatonin treatment on reproductive resumption could be masked by the aforementioned factors. Therefore, the aim of the present research was to evaluate the effect of BCS, treatment period and month of the previous lambing on reproductive resumption in melatonin-treated Sarda breed sheep during spring.

## 2. Materials and Methods

The subjects involved in the research were managed and treated by the farm’s veterinarian who followed the Animal Welfare Organisation’s guidelines and were under the control of the National Health Veterinary Service. The treatments administered to the animals were commonly used techniques performed in sheep farms to improve reproductive performance.

The study was conducted in three farms (1, 2, 3) which were approximately 10 km apart in North Sardinia (40°47′ N, 8°24′ E). Each farm, raising approximately 600 Sarda breed sheep, was under the same pedoclimatic conditions and employed similar feeding and health management routines. The same veterinarians and nutritionists oversaw the three farms. The animals were fed with commercial food at the time of the mechanical milking, had water ad libitum all day, grazed on legumes and gramineous grass during the day and had free access to alfalfa hay during the night.

All of the animals selected for this study were females, with an average age of 4.2 years ± 1.3 and at their third birth. The subjects were identified through the electronic rumen *boli* whose reader was the Allflex RS420 (Allfelx Livestock Intelligence, portable stick reader, Palmerston North, New Zealand). The melatonin treatment consisted of subcutaneous implants being inserted into the left retroauricular region. Each implant contained 18 mg of melatonin Melovine^®^, Ceva Salute Animale, Agrate Brianza, MB, Italy [11]. The melatonin treatment was administered in spring in order to obtain the lambings in autumn. In fact, the characteristic Sardinian sheep productive cycle shows one out-of-season lambing in autumn. This is linked to the Mediterranean climate which is characterized by a mild winter, rainfalls occurring in autumn and spring and a very dry summer. At the time of lambings, sheep exploit the herbage growth cycle and this means that sheep are milked for 8–9 months after lambs are weaned around 30 days. Before the treatment, the rams were separated from the ewes for 40 days. Ram introduction took place 35 days post-treatment. The ratio of males to females was 2:25 for each group. The rams were removed from the groups 50 days after introduction, so that the unmated ewes at this date remained nonpregnant. The veterinarian assessed the rams for their state of health, their fertility and whether they had produced offspring during the previous breeding season. During each trial, all of the groups were raised together and experienced the same management and feeding conditions.

From 150 to 200 days after ram introduction, lambing dates and the number of newborn lambs were registered. From these recorded data, fertility rate (the number of ewes that lambed per ewe exposed to the ram), mean litter size (the number of newborn lambs per ewe lambing) and the distance in days from ram introduction to lambing (DRIL) were calculated. The DRIL was only considered for ewes that lambed.

### 2.1. Trials

#### 2.1.1. First Trial (Different BCS)

The first trial, which was conducted on Farm 1, investigated the effect of melatonin treatment alongside BCS. A total of 100 animals were chosen and divided according to their BCS based upon the methods utilized within Russel et al. [12]. A total of 50 subjects had a BCS of >2.5 (range 2.0–2.5) and the remaining 50 subjects had a BCS of <2.5 (range 2.6–3.0). The animals were further divided into 4 groups as outlined in Table 1.

#### 2.1.2. Second Trial (Different Periods of Treatment)

The second trial, which was conducted on Farm 2, investigated the effect of treatment alongside the date of treatment. All of the sheep within this trial had a previous lambing in November. A total of 150 animals were chosen and divided into 6 groups as outlined in Table 2.

#### 2.1.3. Third Trial (Different Periods of Previous Lambing)

The third trial, which was conducted on Farm 3, investigated the effect of treatment alongside varying dates of the previous lambing. A total of 150 subjects were chosen and divided into 6 groups, as outlined in Table 3.

### 2.2. Statistical Analysis

All the statistical analyses were conducted using R statistical software (Version 4.0.4 R Core Team 2021 R: A language and environment for statistical computing. R Foundation for Statistical Computing, Vienna, Austria; https://www.R-project.org/ accessed on 16 March 2021). To compare the fertility rate amongst each group of every trial, a Chi-square test was used. To evaluate the association between the variables of the first trial (melatonin treatment and BCS) and either DRIL or litter size, a two-way ANOVA and the following linear model was performed:

y_ijk_ = μ + T_i_ + B_j_ + (T_i_B_j_) + S_k_ + e_ijk_ (1)

where y_ijk_ is the trait measured for each animal (DRIL or litter size), Ti is the fixed effect of the treatment (2 levels, j = treated and untreated), B_j_ is the fixed effect of BCS (2 levels, j = >2.5 and <2.5), T_i_B_j_ is the interaction between treatment and BCS, S_k_ is the random effect of the ram (k = 8 rams) and e_ijk_ is the random residual effect of each observation.

To estimate the association between the variables of the second trial (melatonin treatment and the period of treatment) and either DRIL or litter size, a two-way ANOVA and the following linear model was performed:

y_ijk_ = μ + T_i_ + P_j_ + (T_i_P_j_) + S_k_ + e_ijk_ (2)

where y_ijk_ is the trait measured for each animal (DRIL or litter size), T_i_ is the fixed effect of the treatment (2 levels, j = treated and untreated), P_j_ is the fixed effect of the period of treatment (3 levels, j = 15 March, 15 April and 15 May), T_i_P_j_ is the interaction between treatment and the period of treatment, S_k_ is the random effect of the ram (k = 12 rams) and e_ijk_ is the random residual effect of each observation.

To analyse the association between the variables of the third trial (melatonin treatment and the period of previous lambing) and either DRIL or litter size, a two-way ANOVA and the following linear model was performed:

y_ijk_ = μ + T_i_ + L_j_ + (T_i_L_j_) + S_k_ + e_ijk_ (3)

where y_ijk_ is the trait measured for each animal (DRIL or litter size), T_i_ is the fixed effect of the treatment (2 levels, j = treated and untreated), L_j_ is the fixed effect of the period of the previous lambing (3 levels, j = November, December and January), T_i_L_j_ is the interaction between treatment and the period of treatment, S_k_ is the random effect of the ram (k = 12 rams) and e_ijk_ is the random residual effect of each observation.

Data regarding the DRIL and litter size were expressed as least square means ± SEM. Multiple comparisons of the least square means were performed using Tukey’s method (library Agricolae, R package version 1.3-1. Statistical Procedures for Agricultural Research. https://CRAN.R-project.org/package=agricolae, accessed on 25 March 2021). *p* ≤ 0.05 was considered statistically significant.

## 3. Results

### 3.1. First Trial

The statistical analysis showed that the effects of treatment and BCS had a significant influence on the fertility rate. Group A (melatonin-treated sheep with a BCS of >2.5) displayed a higher fertility rate compared to the other groups (*p* < 0.05). Group D (untreated sheep with a BCS of <2.5) exhibited the lowest fertility rate, at 61%. Groups B and C, untreated animals with a BCS of >2.5 and treated animals with BCS of <2.5, respectively, presented with similar fertility rates of 71% and 75%, respectively (Table 4).

No statistical differences were found amongst the groups for litter size (Table 5). However, the effects of melatonin treatment and BCS showed a significant influence on the DRIL (*p* < 0.05, Table 5). Group A exhibited the lowest DRIL (173.29). Additionally, the single effects of treatment and BCS were also evident due to the lower DRIL of Groups B and C compared to Group D.

### 3.2. Second Trial

The statistical analysis showed that the effects of treatment and the period of treatment had a significant influence on the fertility rate. Melatonin-treated animals, particularly those treated in April and May, showed higher fertility rates compared to those who were untreated or treated in March (*p* < 0.001) (Table 6).

Treatment with melatonin implants and the periods of the treatment had no influence on litter size (Table 7). Consequently, all of the groups showed similar litter sizes. On the contrary, melatonin treatment and the period of treatment exhibited a significant effect on DRIL (*p* < 0.05) (Table 7). Melatonin-treated animals evidenced a lower least square means of DRIL (171.67) as compared to the untreated animals (182.82). The same evidence was found for the effect of the period of treatment, where the animals treated in April and May showed a shorter DRIL compared to subjects treated in March (175.71 and 174.12 vs. 181.16).

### 3.3. Third Trial

The statistical analysis showed that the effects of treatment and the period of previous lambing had a significant influence on the fertility rate. Melatonin-treated animals showed a higher fertility rate as compared to untreated animals. Moreover, the treated animals who had their previous lambing in November or December displayed a better fertility rate compared to those who had lambed during the previous January (*p* < 0.05) (Table 8).

Treatment with melatonin implants and the periods of previous lambing had no influence on litter size (Table 9).

In contrast, the effect of treatment positively affected shortened DRIL (*p* < 0.05) (Table 9). Likewise, the effect of the previous lambing also influenced DRIL. Lower DRIL values were observed in sheep that had lambed in the first 15 days (fortnight) of November or December. The animals who were untreated and had their previous lambing in January exhibited a higher DRIL (Table 9).

## 4. Discussion

The findings of the present study were congruent with previously published research [13] and evidenced that melatonin treatment in Sarda breed sheep, via subcutaneous implants, can increase fertility and bring about an advancement in the time of birth. All treatment groups displayed significant effects of treatment.

Another important finding, which was concurrent with our previous studies, was that the litter sizes in Sarda breed sheep were not significantly affected within all trials [7,9]. This aspect remains controversial as certain studies have reported increases in litter size in treated animals [14,15], whilst others have found no effect [16,17,18]. Abecia et al. in (2007) [10], for example, noted an increased litter size after melatonin treatment in Rasa Aragonesa but not in the Assaf breed. Therefore, considering that Sarda and Assaf are both dairy sheep, it could be assumed that some genetic features of these breeds could prevent the effect of melatonin treatment on litter size. The increased fertility rate of the treated sheep compared to the untreated sheep may be linked to the different actions that melatonin performs on the hypothalamus pituitary axis and on the ovary. It has been observed in previous research that melatonin treatment in sheep results in the increased secretion of LH and progesterone. Consequently, this can result in an increased number of embryos and a higher pregnancy rate [19,20]. Furthermore, melatonin has a direct action in the preovulatory follicle due to a higher concentration of melatonin in the follicular fluid than in the bloodstream [21]. Melatonin is involved in the development of the follicle through various actions such as the modulation of the expression of genes that regulate steroidogenesis [22,23], the reduction of the expression of apoptotic genes and the increase of antiapoptotic in granulosa cells [24,25]. Furthermore, the antioxidant melatonin effect at the theca, granulosa and corpus luteum level ensures the protection of follicles and corpus luteum [26,27].

In the first trial, the treated groups showed the best reproductive performance compared to the untreated groups. Furthermore, the BCS displayed an important effect on fertility and DRIL. As observed within Group A, melatonin-treated animals with a BCS of >2.5 exhibited the highest fertility rate and the shortest DRIL compared to the other groups. Together, the melatonin treatment and BCS effects overcame the negative effects of photoperiod on reproductive seasonality. This could be due to the fact that a higher level of BCS is able to positively stimulate the hypothalamus-pituitary-ovary axis, suggesting an interaction between nutritional status and photoperiod [28,29]. This finding was in agreement with the research conducted by Forcada et al. [30], who observed a consistent reduction in the duration of seasonal anestrus in ewes that were constantly kept at a high BCS (2.9) compared to females with a lower BCS (2.3). Therefore, nutritional status can be deemed a significant factor in determining reproductive activity in sheep and the BCS reflects the reliability of this claim [31]. Additionally, some studies in the past have demonstrated that an increase in food intake can induce several ovulations in females and increased sperm production in males [32,33]. Others have corroborated that the signals of metabolic balance can affect the activity of GnRH neurons in the hypothalamus [34]. This is an important point to consider as the neuronal activity of the GnRH is the main controller of gonadal functions. However, several metabolic signals could influence GnRH neurons and, among these, insulin and leptin play an important role in this regulation in sheep [35,36]. Both food intake and level of body reserves affect the circulating concentrations of insulin and leptin, which in turn influence the pulsatile secretion of LH and, consequently, GnRH [35,37]. As a result, a higher BCS could affect the GnRH and LH secretion through metabolic signals which, in combination with the melatonin treatment, could improve the reproductive performance in Group A.

In the second trial treatment, melatonin influenced the reproductive recovery in every treatment period. The best reproductive performances were recorded when the treatment was performed in April and May. This result agrees with what has been found in Europe and in other parts of the world, where melatonin implants have been used to anticipate reproductive activity in spring [6,9,38,39]. Nevertheless, at high latitudes, the administration of melatonin by slow-release implants is usually performed around the summer solstice [40], whereas, in the Mediterranean areas, it is performed around the spring equinox [5]. It is likely that this is linked to the photoperiod trend at different latitudes which also affects the regulation of the reproductive activity of the different sheep breeds living in those areas. In the past decades, several studies were performed to establish the best time period to administer melatonin implants; however, the animal responses were often influenced by the breed and the obtained results were not comparable [8,10]. At the latitudes of the Mediterranean, sheep must be exposed to long days for at least 35 days after a period of decreasing photoperiod in order to be able to stimulate reproductive activity again [41]. Therefore, as seen within this study, low reproductive responses to melatonin in March could be related to a transitional period which is still too close to the deep anestrus. This is congruent with our previous studies, wherein the poorest reproductive results were obtained when the melatonin implants were administered in March [42]. Therefore, by carrying out the treatments in April, a considerable advancement in the reproductive activity is achieved. This allows for the birth to take place at the beginning of autumn, with the possibility of having a fairly long lactation that follows the natural pasture growth pattern [43].

In the third trial, the sheep treated with melatonin implants, that had lambed in the previous November or December, showed the best reproductive performances compared to those who had lambed in January. However, it should be noted that the effect of melatonin was always evident in all treated groups, resulting in better fertility and a shorter DRIL. The lower reproductive efficiency shown by the group of sheep that lambed in January could be due to their lactation phase. This group was in the third month of lactation, during which the milk production is high; Sarda sheep, known for their high milk yield, are subjected to a considerable amount of energy expenditure during milk synthesis. Thus, it is plausible that the vast majority of energy was directed to the mammary gland at the expense of other regions such as the reproductive system. This is concordant with what we found in previous observations, within which the lambs treated with melatonin that have been lambing early have shown a reproductive recovery sooner than those that have been lambing late [9]. To summarize the findings of the last trial, the sheep that were in the first months of lactation were in a period within which the metabolic hormonal asset favours the synthesis of milk but does not favour reproductive activity [44].

## 5. Conclusions

The findings indicate that melatonin is an effective tool for anticipating and improving the reproductive activity of Sardinian sheep during the springtime. Furthermore, to obtain optimal results, melatonin implantation should be conducted in April, in ewes that have a BCS of <2.5 and that have passed their third month of lactation. In the future, this information can be transferred directly to the field and the findings may be used to rationalize the use of melatonin in order to increase reproductive efficiency in sheep farming.

## Figures and Tables

**Table 1:** Groups of the first trial, number of ewes of each group, level of body condition score (BCS) (<2.5 and >2.5), treatment with melatonin implants (treated and untreated) and the date of ram introduction with Sarda breed sheep.

Groups	n. Ewes	BCS	Treatment	Date of Ram Introduction
A	25	>2.5	Treated	20 May
B	25	>2.5	Untreated	20 May
C	25	<2.5	Treated	20 May
D	25	<2.5	Untreated	20 May

**Table 2:** Groups of the second trial, number of ewes of each group, treatment with melatonin implants (treated and untreated), date of treatment (March, April and May) and the date of ram introduction with Sarda breed sheep.

Groups	n. Ewes	Treatment	Date of Treatment	Date of Ram Introduction
E	25	Treated	15 March	19 April
F	25	Untreated	—	19 April
G	25	Treated	15 April	20 May
H	25	Untreated	—	20 May
I	25	Treated	15 May	19 June
L	25	Untreated	—	19 June

**Table 3:** Groups of the third trial, number of ewes of each group, treatment with melatonin implants (treated and untreated), period of previous lambing (November, December and January) and the date of ram introduction with Sarda breed sheep.

Groups	n. Ewes	Treatment	Period of Previous Lambing	Date of Ram Introduction
M	25	Treated	1–15 November	20 May
N	25	Untreated	1–15 November	20 May
O	25	Treated	1–15 December	20 May
P	25	Untreated	1–15 December	20 May
Q	25	Treated	1–15 January	20 May
R	25	Untreated	1–15 January	20 May

**Table 4:** Fertility rate in each group of the first trial, treatment with melatonin implants (treated and untreated) and different body condition scores (BCS) (>2.5 and <2.5).

Groups	Treatment	BCS	Fertility	*p* Value
A	Yes	>2.5	0.82	0.009
B	No	>2.5	0.71	
C	Yes	<2.5	0.75	
D	No	<2.5	0.61	

*p* value ≤ 0.05 was considered statistically significant.

**Table 5:** Experiment 1. Least square means (±SEM) of litter size and days from ram introduction to lambing (DRIL) of the levels of treatment effect (treated and untreated), body condition score (BCS) effect (>2.5 and <2.5) and interaction between treatment and BCS effect with significance of contrasts between least square means in Sarda breed sheep.

Factor	Level	n. Animals	Litter Size	*p* Value	DRIL	*p* Value
Treatment	Treated	50	1.14 ± 0.06	0.366	170.42 ± 10.88 ^a^	0.003
	Untreated	50	1.12 ± 0.05		180.30 ± 10.31 ^b^	
BCS	>2.5	50	1.11 ± 0.06	0.416	172.29 ± 10.55 ^a^	0.043
	<2.5	50	1.15 ± 0.08		178.15 ± 11.99 ^b^	
Treatment by BCS	A ^1^	25	1.15 ± 0.04	0.308	166.40 ± 9.69	0.821
	B ^2^	25	1.10 ± 0.05		176.95 ± 9.89	
	C ^3^	25	1.14 ± 0.04		173.29 ± 10.99	
	D ^4^	25	1.13 ± 0.06		183.83 ± 10.75	

^1^ A = sheep treated with melatonin implants and with a BCS > 2.5; ^2^ B = untreated sheep with a BCS > 2.5; ^3^ C = sheep treated with melatonin implants and with a BCS < 2.5; ^4^ D = untreated sheep with a BCS < 2.5; *p* value ≤ 0.05 was considered statistically significant. Different superscripts within a column (a, b) and within each factor indicate difference for *p* ≤ 0.05.

**Table 6:** Fertility rate in each group of the second trial, treatment with melatonin implants (treated and untreated) and different periods of treatment (March, April and May).

Groups	Treatment	Month of Treatment	Fertility	*p* Value
E	Yes	March	0.71	0.000
F	No	March	0.62	
G	Yes	April	0.84	
H	No	April	0.73	
I	Yes	May	0.83	
L	no	May	0.74	

*p* value ≤ 0.05 was considered statistically significant.

**Table 7:** Experiment 2. Least square means (±SEM) of litter size and days from ram introduction to lambing (DRIL) of the levels of treatment effect (treated and untreated), period of treatment effect (March, April and May) and interaction between treatment and period of treatment effect with significance of contrasts between least square means in Sarda breed sheep.

Factor	Level	n. Animals	Litter Size	*p* Value	DRIL	*p* Value
Treatment	Treated	75	1.14 ± 0.03	0.284	171.67 ± 8.24 ^b^	0.000
	Untreated	75	1.15 ± 0.05		182.82 ± 7.42 ^a^	
Period of treatment	March	50	1.16 ± 0.06	0.512	181.16 ± 9.79 ^a^	0.002
	April	50	1.14 ± 0.07		175.71 ± 12.20 ^b^	
	May	50	1.15 ± 0.05		174.12 ± 9.87 ^b^	
Treatment by period of treatment	E ^1^	25	1.16 ± 0.05	0.359	173.78 ± 10.88	0.077
	F ^2^	25	1.15 ± 0.04		190.64 ± 5.27	
	G ^3^	25	1.13 ± 0.06		172.38 ± 10.03	
	H ^4^	25	1.15 ± 0.05		179.61 ± 8.14	
	I ^5^	25	1.14 ± 0.08		169.14 ± 8.64	
	L ^6^	25	1.15 ± 0.04		179.94 ± 7.95	

^1^ E = sheep treated with melatonin implants in March and exposed to rams on the 19th of April; ^2^ F = sheep untreated in March and exposed to rams on the 19th of April; ^3^ G = sheep treated with melatonin implants in April and exposed to rams on the 20th of May; ^4^ H = sheep untreated in April and exposed to rams on the 20th of May; ^5^ I = sheep treated with melatonin implants in May and exposed to rams on the 19th of June; ^6^ L = sheep untreated in May and exposed to rams on the 19th of June; *p* value ≤ 0.05 was considered statistically significant. Different superscripts within a column (a, b) and within each factor indicate difference for *p* ≤ 0.05.

**Table 8:** Fertility rate in each group of the third experiment and treatment (treated and untreated) with melatonin implants of sheep that lambed in different previous periods (November, December and January).

Groups	Treatment	Previous Lambing	Fertility	*p* Value
M	Yes	1–15 November	0.84	0.002
N	No	1–15 November	0.74	
O	Yes	1–15 December	0.82	
P	No	1–15 December	0.73	
Q	Yes	1–15 January	0.70	
R	No	1–15 January	0.61	

*p* value ≤ 0.05 was considered statistically significant.

**Table 9:** Experiment 3. Least square means (±SEM) of litter size and days from ram introduction to lambing (DRIL) of the levels of treatment effect (treated and untreated), period of the previous lambing effect (November, December and January) and interaction between treatment and period of the previous lambing effect with significance of contrasts between least square means in Sarda breed sheep.

Factor	Level	n. Animals	Litter Size	*p* Value	DRIL	*p* Value
Treatment	Treated	75	1.15 ± 0.04	0.614	171.53 ± 10.96 ^a^	0.000
	Untreated	75	1.13 ± 0.06		184.43 ± 8.22 ^b^	
Period of previous lambing	November	50	1.13 ± 0.05	0.594	175.00 ± 10.62 ^a^	0.000
	December	50	1.16 ± 0.03		173.66 ± 11.70 ^a^	
	January	50	1.14 ± 0.07		182.84 ± 10.24 ^b^	
Treatment by period of previous lambing	M ^1^	25	1.15 ± 0.05	0.274	170.28 ± 10.55	0.550
	N ^2^	25	1.10 ± 0.06		180.50 ± 7.86	
	O ^3^	25	1.15 ± 0.05		166.70 ± 10.61	
	P ^4^	25	1.16 ± 0.05		181.39 ± 7.21	
	Q ^5^	25	1.14 ± 0.03		177.35 ± 9.41	
	R ^6^	25	1.13 ± 0.04		189.06 ± 7.27	

^1^ M = sheep treated with melatonin implants that lambed in the previous November; ^2^ N = sheep untreated that lambed in the previous November; ^3^ O = sheep treated with melatonin implants that lambed in the previous December; ^4^ P = sheep untreated that lambed in the previous December; ^5^ Q = sheep treated with melatonin implants that lambed in the previous January; ^6^ R = sheep untreated that lambed in the previous January; *p* value ≤ 0.05 was considered statistically significant. Different superscripts within a column (a, b) and within each factor indicate difference for *p* ≤ 0.05.

## Data Availability

The data presented in this study are available on request from the corresponding author. The data are not publicly available to preserve privacy of the data.

## References

[1] Chemineau P., Daveau A., Pelletier J., Malpaux B., Karsch F.J., Viguié C. (2003). Changes in the 5-HT2A receptor system in the pre-mammillary hypothalamus of the ewe are related to regulation of LH pulsatile secretion by an endogenous circannual rhythm. BMC Neurosci..

[2] Mura M.C., Luridiana S., Bodano S., Daga C., Cosso G., Diaz M.L., Bini P.P., Carcangiu V. (2014). Influence of melatonin receptor 1A gene polymorphisms on seasonal reproduction in Sarda ewes with different body condition scores and ages. Anim. Reprod. Sci..

[3] Carcangiu V., Mura M.C., Parmeggiani A., Piccione G., Bini P.P., Cosso G., Luridiana S. (2013). Daily rhythm of blood melatonin concentrations in sheep of different ages. Biol. Rhythm. Res..

[4] Malpaux B., Daveau A., Maurice-Mandon F., Duarte G., Chemineau P. (1998). Evidence That Melatonin Acts in the Premammillary Hypothalamic Area to Control Reproduction in the Ewe: Presence of Binding Sites and Stimulation of Luteinizing Hormone Secretion by in Situ Microimplant Delivery 1. Endocrinology.

[5] Chemineau P., Malpaux B., Pelletier J., Leboeuf B., Delgadillo J.A., Deletang F., Pobel T., Brice G. (1996). Use of melatonin implants and photoperiodic treatments to control seasonal reproduction on sheep and goats. INRA Prod. Anim..

[6] De Nicolo G., Parkinson T.J., Kenyon P.R., Morel P.C.H., Morris S.T. (2009). Plasma progesterone concentrations during early pregnancy in spring- and autumn-bred ewes. Anim. Reprod. Sci..

[7] Luridiana S., Mura M.C., Daga C., Farci F., Di Stefano M.V., Zidda F., Carcangiu V. (2015). Melatonin treatment in spring and reproductive recovery in sheep with different body condition score and age. Anim. Reprod. Sci..

[8] Staples L.D., McPhee S., Kennaway D.J., Williams A.H. (1992). The influence of exogenous melatonin on the seasonal patterns of ovulation and oestrus in sheep. Anim. Reprod. Sci..

[9] Carcangiu V., Mura M.C., Bini P.P., Vacca G.M., Daga C., Luridiana S. (2012). Can advance of first lambing induced by melatonin implants influence the next lambing time in Sarda breed sheep?. Can. J. Anim. Sci..

[10] Abecia J.A., Valares J.A., Forcada F., Palacín I., Martín S., Martino A. (2007). The effect of melatonin on the reproductive performance of three sheep breeds in Spain. Small Rumin. Res..

[11] Williams A.H., McPhee S.R., Reeve J.L., Staples L.D. (1992). Optimum use of subcutaneous melatonin implants to enhance the reproductive performance of seasonal and non-seasonal sheep joined in spring and early summer. Anim. Reprod. Sci..

[12] Russel A.J.F., Doney J.M., Gunn R.G. (1969). Subjective assessment of body fat in live sheep. J. Agric. Sci..

[13] Luridiana S., Mura M.C., Daga C., Cosso G., Bodano S., Farci F., Zidda F., Carcangiu V. (2016). Influences of melatonin treatment, melatonin receptor 1A (MTNR1A) and kisspeptin (KiSS-1) gene polymorphisms on first conception in Sarda ewe lambs. Reprod. Fertil. Dev..

[14] Abecia J.A., Forcada F., Zúñiga O. (2002). The effect of melatonin on the secretion of progesterone in sheep and on the development of ovine embryos in vitro. Vet. Res. Commun..

[15] Scott P.R., Sargison N.D., Macrae A.I., Gough M.R. (2009). Melatonin treatment prior to the normal breeding season increases fetal number in United Kingdom sheep flocks. Vet. J..

[16] Rajkumar R.R., Argo C.M., Rodway R.G. (1989). Fertility of ewes given either melatonin or progestogen sponges. Vet. Rec..

[17] Gates P.J., Henningsson T., Tengroth G., Forsberg M. (1998). Effects of Melatonin, Progestagens, and the Ram on Out-of-Season Reproduction in Swedish Landrace Finewool Sheep. Acta Vet. Scand..

[18] Schoeman S.J., Botha M.A. (1995). The effect of melatonin treatment on reproductive efficiency in an accelerated lambing system. J. S. Afr. Vet. Assoc..

[19] Zarazaga L.A., Gatica M.C., Celi I., Guzmán J.L., Malpaux B. (2009). Effect of melatonin implants on sexual activity in Mediterranean goat females without separation from males. Theriogenology.

[20] Song Y., Wu H., Wang X., Haire A., Zhang X., Zhang J., Wu Y., Lian Z., Fu J., Liu G. (2019). Melatonin improves the efficiency of super-ovulation and timed artificial insemination in sheep. PeerJ.

[21] Wang S.J., Liu W.J., Wu C.J., Ma F.H., Ahmad S., Liu B.R., Han L., Jiang X.P., Zhang S.J., Yang L.G. (2012). Melatonin suppresses apoptosis and stimulates progesterone production by bovine granulosa cells via its receptors (MT1 and MT2). Theriogenology.

[22] He C., Ma T., Shi J., Zhang Z., Wang J., Zhu K., Li Y., Yang M., Song Y., Liu G. (2016). Melatonin and its receptor MT1 are involved in the downstream reaction to luteinizing hormone and participate in the regulation of luteinization in different species. J. Pineal Res..

[23] Lima G.N., Maganhin C.C., Simõ Es R.S., Pinheiro Baracat M.C.N., Da Sasso G.R.S., Portugal Fuchs L.F., De Simõ Es M.J., Baracat E.C., Soares Jú Nior J.M. (2015). Steroidogenesis-related gene expression in the rat ovary exposed to melatonin supplementation. Clinics.

[24] Choi J., Park S.M., Lee E., Kim J.H., Jeong Y.I., Lee J.Y., Park S.W., Kim H.S., Hossein M.S., Jeong Y.W. (2008). Anti-apoptotic effect of melatonin on preimplantation development of porcine parthenogenetic embryos. Mol. Reprod. Dev..

[25] Wang S.J., Liu W.J., Wang L.K., Pang X.S., Yang L.G. (2017). The role of Melatonin receptor MTNR1A in the action of Melatonin on bovine granulosa cells. Mol. Reprod. Dev..

[26] Reiter R.J., Rosales-Corral S.A., Manchester L.C., Tan D.X. (2013). Peripheral reproductive organ health and melatonin: Ready for prime time. Int. J. Mol. Sci..

[27] Tamura H., Takasaki A., Taketani T., Tanabe M., Kizuka F., Lee L., Tamura I., Maekawa R., Asada H., Yamagata Y. (2013). Melatonin as a free radical scavenger in the ovarian follicle. Endocr. J..

[28] Forcada F., Abecia J.A. (2006). The effect of nutrition on the seasonality of reproduction in ewes. Reprod. Nutr. Dev..

[29] Menassol J.-B., Collet A., Chesneau D., Malpaux B.B., Scaramuzzi R.J. (2012). The Interaction Between Photoperiod and Nutrition and Its Effects on Seasonal Rhythms of Reproduction in the Ewe 1. Biol. Reprod..

[30] Forcada F., Abecia J.A., Sierra I. (1992). Seasonal changes in oestrus activity and ovulation rate in Rasa Aragonesa ewes maintained at two different body condition levels. Small Rumin. Res..

[31] Bronson F.H. (1998). Energy balance and ovulation: Small cages versus natural habitats. Reprod. Fertil. Dev..

[32] Wade G.N., Schneider J.E. (1992). Metabolic fuels and reproduction in female mammals. Neurosci. Biobehav. Rev..

[33] Martin G.B., Walkden-Brown S.W. (1995). Nutritional influences on reproduction in mature male sheep and goats. J. Reprod. Fertil. Suppl..

[34] Sullivan S.D., DeFazio R.A., Moenter S.M. (2003). Metabolic Regulation of Fertility through Presynaptic and Postsynaptic Signaling to Gonadotropin-Releasing Hormone Neurons. J. Neurosci..

[35] Blache D., Chagas L., Blackberry M., Vercoe P., Martin G. (2004). Metabolic factors affecting the reproductive axis in male sheep. Reproduction.

[36] Barb C.R., Kraeling R.R. (2004). Role of leptin in the regulation of gonadotropin secretion in farm animals. Anim. Reprod. Sci..

[37] Archer Z., Rhind S., Findlay P., Kyle C., Thomas L., Marie M., Adam C. (2002). Contrasting effects of different levels of food intake and adiposity on LH secretion and hypothalamic gene expression in sheep. J. Endocrinol..

[38] Abecia J.A., Palacín I., Forcada F., Valares J.A. (2006). The effect of melatonin treatment on the ovarian response of ewes to the ram effect. Domest. Anim. Endocrinol..

[39] Papachristoforou C., Koumas A., Photiou C. (2007). Initiation of the breeding season in ewe lambs and goat kids with melatonin implants. Small Rumin. Res..

[40] Haresign W., Peters A.R., Staples L.D. (1990). The effect of melatonin implants on breeding activity and litter size in commercial sheep flocks in the UK. Anim. Prod..

[41] Sweeney T., Donovan A., Karsch F.J., Roche J.F., O’Callaghan D. (1997). Influence of previous photoperiodic exposure on the reproductive response to a specific photoperiod signal in ewes. Biol. Reprod..

[42] Mura M.C., Luridiana S., Farci F., Di Stefano M.V., Daga C., Pulinas L., Starič J., Carcangiu V. (2017). Melatonin treatment in winter and spring and reproductive recovery in Sarda breed sheep. Anim. Reprod. Sci..

[43] Carta A., Casu S., Salaris S. (2009). Current state of genetic improvement in dairy sheep. J. Dairy Sci..

[44] Wood R.L., Foster D.L. (1998). Sexual differentiation of reproductive neuroendocrine function in sheep. Rev. Reprod..

